# Overweight modifies the longitudinal association between uric acid and some components of the metabolic syndrome: The Tromsø Study

**DOI:** 10.1186/s12872-016-0265-8

**Published:** 2016-05-10

**Authors:** Jon V. Norvik, Hilde M. Storhaug, Kirsti Ytrehus, Trond G. Jenssen, Svetlana N. Zykova, Bjørn O. Eriksen, Marit D. Solbu

**Affiliations:** Metabolic and Renal Research Group, UiT The Arctic University of Norway, N-9037 Tromsø, Norway; Section of Nephrology, University Hospital of North Norway, N-9038 Tromsø, Norway; Department of Medical Biology, UiT The Arctic University of Norway, N-9037 Tromsø, Norway; Department of Transplant Medicine, Oslo University Hospital Rikshospitalet, N-0424 Oslo, Norway; Centre for Clinical Research and Education, University Hospital of North Norway, N-9038 Tromsø, Norway; Northern Norway Regional Health Authority, N-8038 Bodø, Norway

**Keywords:** Metabolic syndrome, Uric acid, Cardiovascular risk, Overweight, Obesity, Hypertension, Prospective, Cohort, Longitudinal, Insulin resistance

## Abstract

**Background:**

Elevated uric acid (UA) is associated with the presence of the metabolic syndrome (MetS). In a prospective cohort study, we assessed whether baseline and longitudinal change in UA were risk factors for development of MetS and its individual components.

**Methods:**

We included 3087 women and 2996 men who had UA measured in the population based Tromsø Study 1994–95. The participants were stratified according to body mass index (BMI). Endpoints were MetS and each component of the syndrome after 7 years, according to the revised National Cholesterol Education Program’s Adult Treatment Panel III (NCEP-ATP III) definition.

**Results:**

Multiple logistic regression analyses showed that higher baseline UA was associated with higher odds of developing elevated blood pressure in overweight subjects (BMI ≥ 25 kg/m^2^, odds ratio [OR] per 59 μmol/L UA increase 1.44, 95 % confidence interval [CI] = 1.17–1.77, *P* = 0.001), but not in normal-weight subjects (BMI < 25 kg/m^2^, P for interaction = 0.04). Overweight also modified the association between baseline UA and the development of elevated fasting glucose (P for interaction = 0.01). UA was a predictor of MetS in all subjects (OR per 59 μmol/L UA increase 1.29, 95 % CI 1.18–1.41, *P* < 0.001). Furthermore, longitudinal UA change was independently associated with the development of MetS in all subjects (OR per 59 μmol/L UA increase over 7 years 1.28, 95 % CI 1.16–1.42, *P* < 0.001).

**Conclusion:**

Increased levels of baseline UA independently predicted development of elevated blood pressure and higher fasting glycemia in the overweight, but not the normal-weight subjects. Baseline UA and longitudinal increase in UA over 7 years was associated with the development of MetS in all subjects. Whether increased UA should be treated differently in normal-weight and overweight persons needs further study.

## Background

High levels of serum uric acid (UA) are prevalent in the general population. In the National Health and Nutrition Examination Survey (NHANES) 2007–2008 UA levels higher than 339 μmol/L were found in 21.6 % of the women, and among men 21.2 % had UA levels higher than 416 μmol/L [1]. Similar prevalence has been found in China [2]. The incidence and prevalence of hyperuricemia is increasing, as reflected by the increase in the incidence and prevalence of gout since the 1960s [3]. In the US, the prevalence of gout more than doubled between 1969 and 1985 [4], may have increased further over the past two decades [1], and has paralleled a significant increase in prevalence of hyperuricemia [1].

The metabolic syndrome (MetS) is a constellation of interrelated risk factors that increases the risk of cardiovascular disease and type 2 diabetes [5]. MetS is associated with more than two-fold risk of atherosclerotic cardiovascular disease and cardiovascular death [6]. The prevalence of MetS is high in most populations, and in the NHANES 2003–2006 about 34 % of US adults ≥20 years of age fulfilled the MetS definition [7]. One study estimated the worldwide prevalence of MetS to range from <10 % to as much as 84 %, depending on region, sex, age and ethnicity [8]. The prevalence of MetS increased significantly between NHANES 1988–1994 and NHANES 1999–2006, and one of the main reasons for this was the increase in abdominal obesity [9]. Overweight and obesity is an increasing global burden [10] and the number of overweight and obese is projected to continue to grow into the future [11].

UA has been reported to be a risk factor for cardiovascular disease or cardiovascular death in many studies [12–14], but not all [15]. Studies have suggested that hyperuricemia is associated with all of the components of MetS individually: elevated blood pressure [16], obesity [17], high triglycerides [18], low HDL [18] and elevated fasting glucose [19]. Several cross-sectional studies have shown an association between UA and MetS [20, 21], although, after multivariable adjustment, the association disappeared in one study [22]. The role of UA as an independent predictor of the development of MetS has also been examined in several prospective studies. In one study no such association was found [23]. A recent meta-analysis comprising 11 prospective studies concluded that there was an independent, linear dose–response relationship between increasing UA and the development of MetS [24]. As the prevalence of hyperuricemia increases along with the prevalence of overweight and MetS, the causal association between the phenomena remains unsolved. The purposes of the present prospective cohort study were to examine the role of UA and change in UA as a predictor of the MetS and its components after 7 years, and to assess to what extent overweight modified the associations between UA and the metabolic components.

## Methods

### Study population

The Tromsø Study is a series of population-based, prospective surveys of inhabitants of the municipality of Tromsø, Norway [25]. In 1994–1995, 26,969 subjects were investigated (77 % of eligible subjects). Out of these, all participants aged 55–74 years, as well as smaller (5–8 %) random samples of the other age groups <85 years were invited to the more extensive second-visit examination, and 7445 subjects attended (75 % of eligible subjects). Subjects who attended the second visit in 1994–1995 were eligible for the next survey of 2001–2002. In this survey, 6852 subjects who had partaken in the second visit in 1994–1995, participated (89 % of eligible subjects). The number of subjects who died between the two studies was 495. In the present study, we excluded those with missing serum UA analyses (*n* = 405), prevalent diabetes at baseline (*n* = 282; defined as Hba1c ≥6.5 %, non-fasting glucose ≥10.0 mmol/L, under anti-diabetic treatment or self-reported diabetes), and the under-weight subjects (*n* = 82, body mass index [BMI] < 18.5 kg/m^2^). Thus, the final study cohort consisted of 6083 subjects at baseline. The University of Tromsø conducted The Tromsø Study in cooperation with The National Health Screening Service. The Regional Committee for Medical Research Ethics approved the study, and all participants gave their written consent.

### Measurements

All participants provided information on diabetes, alcohol and smoking habits, current use of medication and physical activity through a self-administered questionnaire. Experienced nurses made anthropometric measurements. We calculated BMI as weight (kg)/height (m)^2^. BMI was dichotomized into normal-weight (BMI < 25 kg/m^2^) and overweight (BMI ≥ 25 kg/m^2^). Blood pressure was recorded in triplet after 5-min seating; the mean of the second and third measurement was used. Physical activity was classified as active (≥1 h physical activity with prominent perspiration or breathlessness per week) or inactive (all others). Smoking habits were classified as non-smokers or current smokers. Alcohol intake was classified as teetotalers, 1–7 units/week and >7 units a week. Non-fasting blood samples were drawn and time since last meal was recorded. Serum UA was measured by photometry with COBAS® instruments (Roche diagnostics, Switzerland) using an enzymatic colorimetric test, the uricase/PAP method. Change in UA (ΔUA) was calculated as serum UA in 2001–2002 minus serum UA in 1994–1995. Creatinine was originally analyzed by a modified Jaffe reaction, but because of a possible drift in the results between baseline and follow-up, 111 plasma samples from the 1994–1995 survey and 142 samples from the 2001–2002 survey were thawed and reanalyzed with an enzymatic method (Modular P/Roche Diagnostics) in 2006, as previously described [26]. Values were fitted to a linear regression model, and recalibrated creatinine values were calculated for all participants. Estimated glomerular filtration rate (eGFR) was calculated according to the CKD-EPI formula [27]. Detailed descriptions of measurements of lipids and HbA1c have been published previously [28].

### The metabolic syndrome

Our definition of MetS was based on the revised National Cholesterol Education Program’s Adult Treatment Panel III (NCEP-ATP III) criteria as published by the American Heart Association [5]. Because our data lacked fasting blood samples, we adjusted the definition of elevated triglycerides and elevated glucose. For the definition of elevated fasting glucose, we set the cut off at ≥7.8 mmol/L if time since last meal was under 4 h and at ≥5.6 mmol/L if time since last meal was at least 4 h. For the definition of elevated triglycerides, we set the cut-off at ≥2.28 mmol/L if time since last meal was under 4 h, as non-fasting triglyceride levels are on average 20 to 30 % higher than fasting levels [29], and ≥1.7 mmol/L if time since last meal was at least 4 h. Thus, the definition of MetS in this study is any three (or more) out of the following five criteria: increased waist circumference (≥88 cm in women and ≥102 cm in men), elevated triglycerides (triglycerides ≥1.7 mmol/L if time since last meal ≥4 h, ≥ 2.28 mmol/L if time since last meal <4 h or use of lipid-lowering drugs), reduced HDL-cholesterol (HDL < 1.30 mmol/L in women and <1.03 mmol/L in men), elevated blood pressure (≥130 mm Hg systolic blood pressure, ≥ 85 mm Hg diastolic blood pressure or antihypertensive drug treatment) and elevated fasting glucose (glucose ≥ 5.6 mmol/L if time since last meal ≥4 h, ≥7.8 mmol/L if time since last meal <4 h or on treatment for elevated glucose).

### Statistics

Data are given as mean ± standard deviation (SD). Independent sample t-tests and chi square tests were applied to compare baseline variables between participants with normal-weight and overweight. In each of these groups, we assessed Pearson’s correlation coefficient between UA and the baseline variables. We conducted multiple binary logistic regression analyses with each single criterion of MetS and MetS (any three criteria or more) in 2001–2002 as dependent variables and uric acid as an independent variable in separate models. Covariates were sex, age, systolic blood pressure, total cholesterol, current smoking, physical activity, Hba1c, eGFR, alcohol consumption, use of diuretics and waist circumference at baseline. In each of these analyses, we only included the subjects who did not fulfill the MetS criterion of interest at baseline. We ran the analyses both with the entire cohort and stratified into normal-weight and overweight (BMI < 25 kg/m^2^ and BMI ≥ 25 kg/m^2^) at baseline, and interaction between UA and the BMI group was tested for. We also checked for interactions between UA and gender and UA and a BMI-cutoff of obesity (BMI < 30 kg/m^2^ and BMI ≥ 30 kg/m^2^) for MetS and each of the MetS criteria. The logistic regression analyses were repeated for the group of subjects who did not have MetS at baseline. Finally, we assessed whether ΔUA was associated with MetS and its components by adding ΔUA as an independent variable to each model. These analyses were also run in the group without MetS at baseline. Two-sided *P* values < 0.05 were considered statistically significant. We did all the analyses using SPSS software version 22.0 (IBM Corp. Released 2013. IBM SPSS Statistics for Windows, Version 22.0. Armonk, NY: IBM Corp).

## Results

### Baseline characteristics

Baseline characteristics of the cohort divided into normal-weight (BMI < 25 kg/m^2^) and overweight (BMI ≥ 25 kg/m^2^) are shown in Table 1. The differences between the two groups were statistically significant for all the variables. Being overweight, compared to normal-weight, was associated with male gender, older age, and generally a more adverse cardiovascular risk profile, including higher blood pressure, lower eGFR and a poorer lipid profile. On the other hand, there was a larger proportion of smokers in the normal-weight stratum. Also shown in Table 1 is the correlation between each variable and UA. In addition to gender, waist circumference, triglycerides, HDL and MetS correlated strongest with UA, whereas eGFR, use of diuretics, alcohol consumption and blood pressure correlated weaker with UA. The rest of the variables displayed a very weak correlation with UA or no correlation at all. Median time since last meal at baseline was between 2 and 3 h, 16.6 % had at least 4 h since last meal, and 6.1 % had at least 8 h since last meal. In this cohort, 57.9 % of the subjects were overweight (BMI ≥ 25 kg/m^2^) and 13.5 % were obese (BMI ≥ 30 kg/m^2^).

**Table 1:** Cohort characteristics according to classification by body mass index (BMI) definition of normal-weight/overweight

		BMI < 25 kg/m^2^, *n* = 2556				BMI ≥ 25 kg/m^2^, *n* = 3527				
			SD/%	Pearson correlation with UA	*P* for Pearson correlation		SD/%	Pearson correlation with UA	*P* for Pearson correlation	*P* between groups of BMI cut-off
Age, years		58.8	±11.3	0.09	<0.001	60.9	±9.1	−0.08	<0.001	<0.001
Sex	Men	1149	45.0 %	0.51	<0.001	1847	52.4 %	0.44	<0.001	<0.001
	Women	1407	55.0 %			1680	47.6 %			
Systolic blood pressure, mm Hg		139.5	±21.7	0.11	<0.001	148.3	±22.1	0.03	0.046	<0.001
Waist circumference, cm	Men	87.7	±5.6	0.45	<0.001	99.5	±7.6	0.39	<0.001	<0.001
	Women	77.5	±6.3			91.0	±9.5			
Serum HDL, mmol/L		1.65	±0.45	−0.28	<0.001	1.46	±0.40	−0.35	<0.001	<0.001
Uric acid μmol/L	Men	334.1	±72.9	–	–	377.7	±88.4	–	–	<0.001
	Women	254.5	±63.4	–	–	298.9	±72.0	–	–	<0.001
Triglycerides, mmol/L		1.27	±0.67	0.45	<0.001	1.73	±0.98	0.55	<0.001	<0.001
Plasma glucose, mmol/L		4.64	±0.58	0.03	0.094	4.82	±0.62	0.11	<0.001	<0.001
HBA1C, %		5.36	±0.35	0.03	0.229	5.42	±0.37	0.05	0.003	<0.001
Total cholesterol, mmol/L		6.56	±1.33	0.07	<0.001	6.89	±1.25	0.01	0.666	<0.001
eGFR, mL/min/1.73 m^2^		94.6	±13.6	−0.23	<0.001	91.6	±13.0	−0.14	<0.001	<0.001
Use of diuretics, *n*		23	0.9 %	0.12	<0.001	78	2.2 %	0.13	<0.001	<0.001
Use of allopurinol, *n*		0	–	–	–	15	0.4 %	0.03	0.113	0.001
Daily smoker, *n*		1026	40.1 %	−0.01	0.503	935	26.5 %	0.03	0.131	<0.001
Alcohol consumption, teetotalers, *n*		1277	50.0 %	0.14	<0.001	1936	54.90 %	0.19	<0.001	0.001
Alcohol consumption, 1–7 units/week, *n*		1186	46.4 %	–	–	1435	40.70 %	–	–	–
Alcohol consumption, > 7 units/week, *n*		93	3.6 %	–	–	156	4.40 %	–	–	–
Physical activity, *n*		608	23.8 %	0.04	0.044	720	20.4 %	0.03	0.086	0.002
Elevated blood pressure, *n*		1696	66.4 %	0.14	<0.001	2943	83.4 %	0.11	<0.001	<0.001
Central obesity, *n*		95	3.7 %	−0.04	0.058	1631	46.2 %	0.06	<0.001	<0.001
Elevated triglycerides, *n*		252	9.9 %	0.33	<0.001	894	25.3 %	0.41	<0.001	<0.001
Low HDL, *n*		282	11.0 %	0.05	0.007	724	20.5 %	0.18	<0.001	<0.001
Elevated fasting glucose, *n*		15	0.6 %	0.01	0.666	65	1.8 %	0.03	0.104	<0.001
Metabolic syndrome, *n*		91	3.6 %	0.20	<0.001	788	22.3 %	0.32	<0.001	<0.001

The first column in each strata provides means for the continuous variables and numbers for the categories

Elevated blood pressure = blood pressure ≥130/85 mm Hg or treated for hypertension, elevated triglycerides = triglycerides ≥2.28 mmol/L if time since last meal <4 h and ≥1.7 mmol/L if time since last meal ≥4 h or use of lipid lowering drugs, low HDL = HDL < 1.03 mmol/L in men or <1.30 mmol/L in women, elevated fasting glucose = glucose ≥ 7.8 mmol/L if time since last meal <4 h and ≥5.6 mmol/L if time since last meal ≥4 h or treated for elevated glucose, central obesity = waist circumference ≥ 102 cm in men or ≥88 cm in women, MetS = three or more MetS components

*Abbreviations*: *BMI* body mass index, *UA* uric acid, *SD* standard deviation, *HDL* high-density lipoprotein, *HBA1c* hemoglobin A1c, *eGFR* estimated glomerular filtration rate

### Associations between baseline UA and subsequent changes in the components of MetS stratified by BMI

**Fig. 1:**
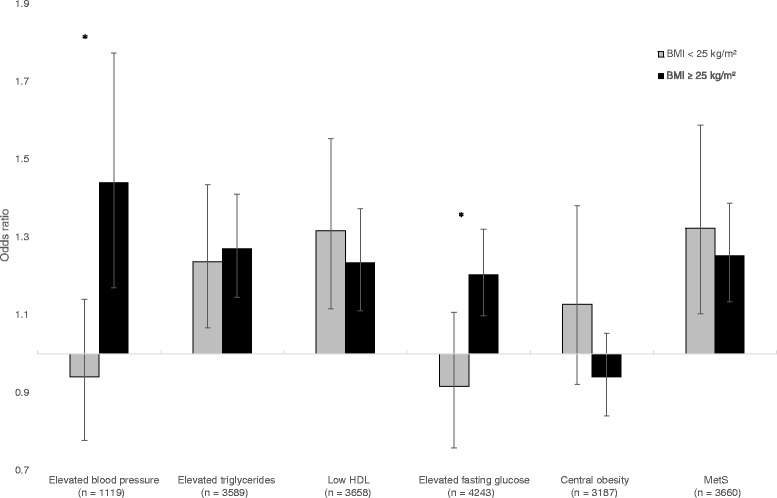
Multivariable logistic regression with baseline serum uric acid (UA) as predictor of the metabolic syndrome and its components after 7 years, stratified into normal-weight and overweight at baseline by body mass index (BMI). The odds ratio is per 59 μmol/L increase of UA. *Bars* represent odds ratio; *grey bars* the normal-weight subjects (BMI < 25 kg/m^2^) and *black bars* the overweight subjects (BMI ≥ 25 kg/m^2^). *Whiskers* represent 95 % confidence interval. The group includes the subjects without each component of MetS of interest or MetS (three or more components) at baseline. Covariates: baseline sex, age, systolic blood pressure, total cholesterol, current smoking, physical activity, HbA1c, eGFR, alcohol consumption, use of diuretics, and waist circumference. * = P for interaction with BMI-cut-off <0.05. Elevated blood pressure: blood pressure ≥130/85 mm Hg or treated for hypertension, elevated triglycerides: triglycerides ≥2.28 mmol/L if time since last meal <4 h and ≥1.7 mmol/L if time since last meal ≥4 h or use of lipid lowering drugs, low HDL = HDL < 1.03 mmol/L in men or <1.30 mmol/L in women, elevated fasting glucose = glucose ≥7.8 mmol/L if time since last meal <4 h and ≥5.6 mmol/L if time since last meal ≥4 h or treated for elevated glucose, central obesity = waist circumference ≥102 cm in men or ≥88 cm in women, MetS = three or more MetS components. Abbreviations: *HDL* high-density lipoprotein, *BMI* body mass index, *MetS* metabolic syndrome, *UA* uric acid, *HBA1c* hemoglobin A1c, *eGFR* estimated glomerular filtration rate

Figure 1 displays the multivariable adjusted odds ratio (OR) of 59 μmol/L (1 mg/dL) UA increase at baseline for acquiring each component of MetS and MetS (three or more components of MetS) after 7 years, stratified by baseline BMI. Each outcome was assessed in the subjects who did not fulfill the criteria for the outcome of interest at baseline. Among the normal-weight individuals, 669 subjects were normotensive at baseline according to the MetS criteria. After 7 years, 251 of them had developed elevated blood pressure. Baseline UA was not a predictor of elevated blood pressure in this group. Among the 450 overweight subjects who were normotensive at baseline, 227 had developed elevated blood pressure 7 years later. Baseline UA was an independent predictor of elevated blood pressure in the overweight (OR per 59 μmol/L UA 1.44, 95 % confidence interval [CI] 1.17–1.77, *P* = 0.001). The interaction between the BMI-cutoff and UA for the prediction of new cases of elevated blood pressure was significant (*P* = 0.04). There were no statistically significant interactions between neither UA and gender nor UA and a BMI-cutoff of obesity (BMI < 30 kg/m^2^ and BMI ≥ 30 kg/m^2^) for any of the outcomes.

BMI also modified the association between UA at baseline and new cases of elevated fasting glucose after 7 years (Fig. 1). In the overweight, baseline UA predicted this outcome (OR per 59 μmol/L UA increase 1.20, 95 % CI = 1.10–1.32, *P* < 0.001), whereas baseline UA was not significantly associated with new onset fasting glucose elevation for subjects with normal-weight (P for interaction = 0.01). There was no interaction between UA and the BMI cut-off for elevated triglycerides and low HDL cholesterol according to MetS criteria (P for interaction = 0.39 for both), and UA did not significantly predict the development of central obesity according to the MetS criteria in either group.

### The association between UA and new cases of MetS

Results of unstratified multivariable logistic regression analyses of the subjects without MetS at baseline are displayed in Table 2. In this group, 611 subjects had MetS 7 years later, and baseline UA was a predictor of this outcome (OR per 59 μmol/L increase 1.29, 95 % CI = 1.18–1.41, *P* < 0.001).

**Table 2:** Multivariable logistic regression with baseline serum uric acid (UA) as a predictor of the metabolic syndrome (MetS) and its components after seven years, unstratified. The odds ratio (OR) is per 59 μmol/L increase of UA. The group includes the subjects without MetS at baseline

	Number	Cases	OR	95 % CI	*P* value
Risk of elevated blood pressure	3701	2847	1.15	[1.04–1.27]	0.006
Risk of elevated triglycerides	3693	941	1.32	[1.22–1.42]	<0.001
Risk of low HDL	3690	553	1.27	[1.16–1.39]	<0.001
Risk of elevated fasting glucose	3689	333	1.13	[1.02–1.26]	0.021
Risk of central obesity	3677	1031	1.07	[0.97–1.18]	0.130
Risk of MetS	3660	611	1.29	[1.18–1.41]	<0.001

Covariates: sex, age, systolic blood pressure, total cholesterol, current smoking, physical activity, HbA1c, eGFR, use of diuretics, alcohol consumption, and waist circumference

Elevated blood pressure = blood pressure ≥ 130/85 mm Hg or treated for hypertension, elevated triglycerides = triglycerides ≥ 2.28 mmol/L if time since last meal <4 h and ≥1.7 mmol/L if time since last meal ≥4 h or use of lipid lowering drugs, low HDL = HDL < 1.03 mmol/L in men or <1.30 mmol/L in women, elevated fasting glucose = glucose ≥ 7.8 mmol/L if time since last meal <4 h and ≥5.6 mmol/L if time since last meal ≥4 h or treated for elevated glucose, central obesity = waist circumference ≥ 102 cm in men or ≥88 cm in women, MetS = three or more MetS components

*Abbreviations*: *UA* uric acid, *OR* odds ratio, *BMI* body mass index, *CI* confidence interval, *HDL* high-density lipoprotein, *HBA1c* hemoglobin A1c, *eGFR* estimated glomerular filtration rate, *MetS* metabolic syndrome

### Change in UA as a risk factor for MetS and its components

Change in UA over 7 years as a predictor of MetS and its single components in 2001–2002 was assessed in multiple logistic regression models. The results are shown in Table 3. An increase in UA by 59 μmol/L over 7 years from baseline implied an increase in odds of MetS of 28 %. UA increase was also an independent risk factor for new cases of elevated blood pressure, elevated triglycerides, low HDL and central obesity. However, longitudinal UA increase was not a risk factor for incident elevated fasting glucose.

**Table 3:** Multivariable logistic regression with longitudinal serum uric acid change (ΔUA) from baseline to seven years later as a predictor of the metabolic syndrome and its components after seven years. The odds ratio (OR) is per 59 μmol/L UA increase. The group includes the subjects without MetS at baseline

	Number	Cases	OR	95 % CI	*P* value
Risk of elevated blood pressure	3507	2693	1.16	[1.02–1.31]	0.021
Risk of elevated triglycerides	3507	888	1.20	[1.10–1.31]	<0.001
Risk of low HDL	3506	528	1.18	[1.07–1.31]	0.001
Risk of elevated fasting glucose	3506	320	0.97	[0.86–1.10]	0.636
Risk of central obesity	3484	978	1.49	[1.33–1.66]	<0.001
Risk of MetS	3477	586	1.28	[1.16–1.42]	<0.001

Covariates: sex, age, systolic blood pressure, total cholesterol, current smoking, physical activity, HbA1c, eGFR, alcohol consumption, use of diuretics, waist circumference, baseline UA

Elevated blood pressure = blood pressure ≥ 130/85 mm Hg or treated for hypertension, elevated triglycerides = triglycerides ≥ 2.28 mmol/L if time since last meal <4 h and ≥1.7 mmol/L if time since last meal ≥4 h or use of lipid lowering drugs, low HDL = HDL < 1.03 mmol/L in men or <1.30 mmol/L in women, elevated fasting glucose = glucose ≥ 7.8 mmol/L if time since last meal <4 h and ≥5.6 mmol/L if time since last meal ≥4 h or treated for elevated glucose, central obesity = waist circumference ≥ 102 cm in men or ≥88 cm in women, MetS = three or more MetS components

*Abbreviations*: *ΔUA* uric acid change, *UA* uric acid, *OR* odds ratio, *BMI* body mass index, *CI* confidence interval, *HDL* high-density lipoprotein, *HBA1c* hemoglobin A1c, *eGFR* estimated glomerular filtration rate, *MetS* metabolic syndrome

## Discussion

In this large prospective study of subjects without diabetes from the general population, elevated UA at baseline was independently associated with increased risk of elevated blood pressure in the overweight individuals 7 years later. We found no association between UA and future elevated blood pressure in the normal-weight subjects. Moreover, UA at baseline predicted new-onset impaired fasting glucose in the overweight persons, but not in the normal-weight group. Baseline UA was a predictor of MetS in all subjects. Finally, a longitudinal increase in UA of 59 μmol/L over 7 years raised the odds of developing MetS by 28 %.

The association between UA and MetS is in accordance with previous prospective studies [17, 30, 31]. Few studies have examined the association between longitudinal UA change and MetS. In a healthy Japanese cohort, no significant association was found between 1 mg/dL (59 μmol/L) UA increase and incident MetS [32]. However, in the Japanese study, follow-up time was shorter than in our study, and the authors did not adjust for baseline UA. These methodological differences may in part explain the discrepancies between the results of our study and the study from Japan.

To the best of our knowledge, there are no other studies of this scale where the population is stratified into normal-weight/overweight before examining the association between UA and MetS and its components. A small study (*n* = 69) from the United Arab Emirates examined the univariable relationship between a set of biomarkers, among them UA, and components of MetS in healthy, young females, stratified into normal-weight (BMI ≤ 25 kg/m^2^), overweight (BMI > 25, < 30 kg/m^2^), and obese (BMI ≥ 30 kg/m^2^) [33]. This study found statistically significant correlations between UA and the waist circumference and triglycerides components only, and the associations were confined to the obese group. The authors found no significant correlation between UA and blood pressure in the strata; this may be due to small sample size and a population of uniform age and sex. In our study, we did not find any statistically significant interaction between the BMI-cutoff of obesity (BMI < 30 kg/m^2^ and BMI ≥ 30 kg/m^2^) for neither MetS nor any of its components. This may be due to a small group of obese in our cohort.

The association between hypertension and UA was first noted in the 1870s and has been demonstrated in numerous publications. In a recent meta-analysis, UA increase was reported to be associated with a statistically significant elevation in incident hypertension [16]. It has been claimed that an elevated serum UA is the independent risk factor for hypertension that is the most reproducible to date [34]. A multitude of studies, in an effort to explain how hyperuricemia can lead to hypertension and cardiovascular disease, have proposed interlinked mechanisms such as endothelial dysfunction and reduction in endothelial nitric oxide (NO) levels [35], oxidative stress [36], activation of the renin-angiotensin-aldosterone-system (RAAS) [37] and renal microvascular lesions [38]. However, we found that UA was a predictor of elevated blood pressure in the overweight, but not in the normal-weight. Few studies have explored this phenomenon. The precursor of UA is xanthine, and the reaction from the latter to the former is catalyzed by the enzyme xanthine oxidoreductase (XOR), which can exist in two forms, xanthine dehydrogenase (XDH) or xanthine oxidase (XO) [39]. The enzyme is mostly in its XDH form, but can be transformed into XO by proteolytic cleavage or oxidation. In the XO form, reactive oxygen species are a by-product of the reaction of xanthine to UA [40]. Therefore, under certain circumstances, increased activity of XO, detected as elevated production of UA, will lead to increased oxidative stress, which, in turn, can be detrimental in the state of reduced antioxidant capacity that accumulated fat creates [41]. Furthermore, UA can affect adipocytes by inducing upregulation of pro-inflammatory factors and downregulation of the insulin sensitizer and anti-inflammatory factor adiponectin [42]. Adiponectin is negatively associated with BMI and body-fat [43]. Since low levels of adiponectin is associated with the development of hypertension [44] and insulin resistance [45], it could be speculated that adiponectin is part of the link between UA and elevated blood pressure and insulin resistance, and explain why UA is associated with new onset elevated blood pressure and impaired fasting glucose in the overweight but not the normal-weight in our study. Furthermore, a study found increased angiotensinogen levels in the hypertensive overweight (BMI ≥ 25 kg/m^2^), compared to the hypertensive normal-weight (BMI < 25 kg/m^2^), in the presence of hyperuricemia [46], and a rodent model demonstrated that UA-mediated upregulation of adipose RAAS caused insulin resistance [47]. UA might also directly contribute to the development of insulin resistance in adipose tissue, possibly through redox modulation [48]. These could also be mechanisms in which UA is associated with overweight-related elevated blood pressure and elevated fasting glucose.

Epidemiologically, UA is associated with insulin resistance [49], and the development of insulin resistance is often preceded by hyperuricemia [50]. MetS does not comprise a uniform group of subjects; Sperling et al. of The Cardiometabolic Think Tank present a subtype where insulin resistance is dominant [51]. An association between hyperuricemia and insulin resistance could in part explain the development of MetS.

The present study has important strengths: the large size, solid attendance rate, long follow-up time, use of UA as a continuous variable, and the ability to correct for confounders such as eGFR, use of diuretics and all the traditional cardiovascular risk factors. However, a major shortcoming of our study is the lack of fasting blood samples. In particular, glucose and triglycerides, and thereby the definition of MetS, are affected by this. The incorporation of time since last meal and adjustment of the cut-offs in the definition of elevated fasting glucose and elevated triglycerides compensated in part, but not fully, for this limitation. In addition, only a single measurement of serum UA was done in each survey. Another shortcoming of this study may be the fact that our baseline data were collected 21–22 years ago, and 14–15 years have passed since follow-up. Both lifestyles and pharmacological treatment have changed in that time. However, if the effects of overweight on UA’s association with MetS can be reproduced in studies on newer data, our findings may be even more relevant as overweight and obesity is an even greater challenge in the world of today. That our study population comprised largely of healthy, middle-aged to elderly Caucasians can be viewed as both a weakness and a strength; the results may not be generalizable to dissimilar populations, but the homogeneity of our cohort may have prevented dilution of our findings due to important diversities in baseline properties.

## Conclusion

In a large cohort from the general population, baseline UA was independently associated with future cases of elevated blood pressure and elevated fasting glucose in overweight subjects, but not normal-weight individuals. Both elevated baseline UA and longitudinal increase in UA over 7 years from baseline was associated with the development of MetS in all subjects. These findings warrant further studies to examine the exact causal relationship between UA and MetS, in overweight as well as in normal-weight individuals, and to assess whether treatment strategies need to be targeted differentially according to BMI.

### Ethics approval and consent to participate

The Regional Committee for Medical Research Ethics approved the study (committee’s reference number 2009/2536-3), and all participants gave their written consent to participate. The Tromsø Study complies with the Declaration of Helsinki.

### Consent for publication

Not applicable.

### Availability of data and materials

The dataset supporting the conclusions of this article is legally restricted. It will therefore not be shared publicly. However, meta-data as well as general information about the study is available at http://tromsoundersokelsen.uit.no/tromso.

## Abbreviations

BMI: body mass index
CI: confidence interval
CKD-EPI: The Chronic Kidney Disease Epidemiology Collaboration
eGFR: estimated glomerular filtration rate
HBa1c: hemoglobin A1c
HDL: high-density lipoprotein
MetS: the metabolic syndrome
NCEP-ATP III: National Cholesterol Education Program’s Adult Treatment Panel III
NHANES: National Health and Nutrition Examination Survey
NO: nitric oxide
OR: odds ratio
RAAS: renin-angiotensin-aldosterone-system
SD: standard deviation
UA: uric acid
ΔUA: change in quantity of uric acid
